# PPG-Terminated Tetra-Carbamates as the Toughening Additive for Bis-A Epoxy Resin

**DOI:** 10.3390/polym11091522

**Published:** 2019-09-19

**Authors:** Ming Zhang, Mingqing Chen, Zhongbin Ni

**Affiliations:** School of Chemical and material engineering, Jiangnan University, Wuxi 214122, China; thhy107@126.com (M.Z.); polymer@jiangnan.edu.cn (Z.N.)

**Keywords:** tetra-carbamates, epoxy polymer, toughening, microphase separation, particle

## Abstract

We synthesized PPG-terminated tetra-carbamates as a new toughening additive for epoxy thermosets through facile addition reaction of hexamethylene diisocyanate (HDI) with poly(tetra-methylene glycols) (PTMG) and poly(propylene glycols) (PPG). The effects of prepared tetra-carbamates on the rheological behavior of neat epoxy resin were studied along with the various cured properties of their modified epoxy systems. Four carbamate groups (–NHCOO–) endow the prepared additives not only with good intramolecular interactions, but also with optimal intermolecular interactions with epoxy polymers. This results in the suitable miscibility of the additives with the epoxy matrix for the formation of the typical biphasic structure of microparticles dispersed in the epoxy matrix via polymerization-induced microphase separation. The impact strength and critical stress concentration factor (*K*_IC_) of cured modified epoxy systems with the additives are significantly higher than those of unmodified epoxy systems, without sacrificing the processability (*T*_g_) and flexural strength. The toughening mechanism is understood as a synergism combination among the phase separation mechanism, the in situ homogeneous toughening mechanism, and the particle cavitation mechanism.

## 1. Introduction

Epoxy resins represent the most common class of thermosetting materials. Their highly crosslinked microstructures endow them with many useful properties for industrial applications, for instance, high strength, excellent thermal stability, and good adhesion. Nevertheless, their resistance to crack initiation and propagation is poor due to their inherent brittleness, which affects their durability and strongly constrains their design parameters [1,2,3,4]. Thus, toughening epoxy resin has been a major research topic over the last three decades. The most common modifying strategy is to introduce a second dispersed fine phase into the epoxy polymers, through which the toughness of epoxy polymers can be significantly improved without impairing other desirable engineering properties, such as the commercial carboxy-terminated polybutadiene (CTBN), hydroxyl-terminated polyurethane, and hyperbranched polymers [5,6,7,8,9].

Block copolymers (BCs) have attracted considerable attention through the toughening of epoxy polymers via the ordered micro/nanostructures since Hillmyer et al. first reported the ordered nanostructures within thermosets modified with BCs in 1977 [10]. BCs can self-assemble into various ordered micro/nanostructures due to their interesting amphiphilic behaviors. Poly(ethylene oxide)-poly (propylene oxide)-poly(ethylene oxide) (PEO-PPO-PEO), PPO-PEO, and poly(ethylene oxide-b-ε-caprolactone) (PEO-b-PCL) represent the most popular BCs employed as toughening additives for epoxy resins due to their fascinating amphiphilic characteristics and commercial availabilities [11,12,13,14]. These copolymers get their amphiphilic characteristics with PEO blocks miscible with epoxy matrix from the main driving force of hydrogen bonding interaction, and PPO blocks immiscible with epoxy matrix, which endows them with the self-assembly behaviors and reaction-induced microphase separation in epoxy systems [11,12,13,14]. Thus, understanding the interaction and miscibility between components becomes the key to achieve the ordered morphology in high-performance epoxy polymers. There is a large amount of literature on the subject of mediating the interaction and miscibility of PEO blocks with the epoxy matrix to get the ordered morphology through the appropriate design of the molecules of BCs, such as the PEO/PPO ratio, reducing molecular weight, introducing epoxy-phobic alkane blocks, and epoxidizing the block [11,14,15,16].

However, little has been published on introducing carbamates of –NHCOO– as the molecular segments of additives to mediate the interaction and miscibility of modifiers with the epoxy matrix to achieve the ordered micro/nanostructures. Herein, the tetra-carbamates with four hydrogen bond motifs separated by one poly(tetra-methylene glycols) (PTMG) segment and having two terminal poly(propylene glycols) (PPG) chains were firstly synthesized through the facile addition reaction of the commercial materials of HDI, PTMG, and PPG. The average molecular weights of up to 2000 g/mol were preferred for both polyether glycols considering the processability of modified epoxy with the prepared tetra-carbamates [17]. The cured modified epoxy systems with tetra-carbamates exhibit similar biphasic micro/nanostructures in the epoxy matrix to those reported in the modified epoxy systems with BCs, such as PEO-PPO-PEO and PEO-b-PCL [11,12,13,14,15,16]. Accordingly, the toughness of the modified epoxy systems with the prepared tetra-carbamates is significantly improved without sacrificing processability (*T*_g_) and mechanical strength, even at the lower contents of additives of 2 and 5 wt %. The intramolecular and intermolecular interactions between the related components arising from the significant hydrogen bonding are proposed to be the main driving force for the ordered microphase separation and prominent toughness improvement of modified epoxy polymers with the tetra-carbamates.

## 2. Materials and Methods 

### 2.1. Materials

Hexamethylene diisocyanate (HDI, AR) and triethylenetetramine (TETA) were purchased from Aladdin Industrial Corporation (Shanghai, China) and used as received. PTMG (PTMG-650 and PTMG-2000, whose average molecular weight is 650 and 2000 g/mol, respectively), PPG (PPG400, whose average molecular weight is 400 g/mol.), *N,N*-dimethylformamide (DMF, AR) and acetone (AR) were purchased from Aladdin Industrial Corporation and dried by 3A molecular sieves for one week. Organic tin (Thermolite 890) was obtained from PMC Group (Beijing, China) and used as received. Bisphenol-A epoxy resin whose epoxide equivalent is about 189g/mol (DER331), was purchased from DOW (Zhangjiagang, China) and used as received.

### 2.2. Synthesis of PPG-Terminated Tetra-Carbamates

**Figure 1:**
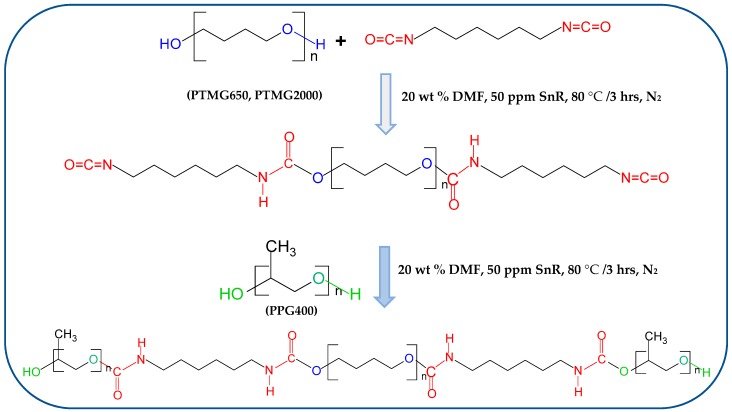
Synthetic pathway of tetra-carbamates.

The preparation of the tetra-carbamates was performed in a 250 mL three-necked glass reactor. Firstly, a certain amount of DMF, HDI, and Thermolite 890 was added into the reactor and stirred by a mechanical stirrer for 15 min under N_2_ atmosphere at 80 °C. Then a weighed amount of PTMG dissolved in DMF was added dropwise into the reactor within 3 h under N_2_ atmosphere at 80 °C. The molar ratio of NCO to OH was fixed at 2.05/1.00. A transparent DMF solution containing NCO–terminated PTMG was obtained for the next synthesis reactions. Secondly, a certain amount of DMF, PPG400, and Thermolite 890 was added into another reactor and stirred by a mechanical stirrer for 15 min under N_2_ atmosphere at 80 °C. A weighed amount of DMF solution containing NCO–terminated PTMG was added dropwise into the reactor within 3 h under N_2_ atmosphere at 80 °C. The molar ratio of NCO to OH was fixed at 1.00/2.05. The reaction concentration of 20 wt % was employed for all synthesis reactions, and Thermolite 890 was controlled at 0.005 wt % for all synthesis reactions. The synthetic pathway is shown in Figure 1. The reaction products were purified by a massive solution of water and acetone, and dried in a vacuum oven at 80 °C for 3 days. The name of the tetra-carbamates sample was abbreviated as shown in Table 1 according to the average molecular weight of composed PTMG.

### 2.3. Preparation of Modified Epoxy Resin with Tetra-Carbamates

The tetra-carbamates sample was introduced into DER331 at different mass percentages under constant stirring for 15 min at 60 °C in a vacuum condition until a homogeneous blend was achieved. The obtained modified epoxy samples were cooled down to 25 °C. Subsequently, the modified epoxy resins were mixed with TETA through centrifugal mixing for 2 min in a vacuum condition at the mass ratio of DER331/TETA = 100:15. The final epoxy ternary mixtures obtained were cast into stainless molds in a vacuum, and were subjected to the following curing procedures: firstly cured at 60 °C for 2 h, and then post-cured at 130 °C for 2 h for the complete curing. Finally, the cured modified epoxy samples were naturally cooled down to room temperature.

### 2.4. Characterization

An FT-IR Spectrophotometer (Prestige-21, SHIMATSU, Kyodo, Japan) was employed to record the infrared spectra of samples. The KBr tablet with the sample was scanned from 4000 to 300 cm^−1^. The spectra were obtained as percentage of transmittance vs. wavenumber. Thermal properties of samples were analyzed by a differential scanning calorimeter (DSC7020, HITACHI, Tokyo, Japan) at a temperature ramping rate of 5 °C/min in a nitrogen atmosphere. The rheological properties of various uncured epoxy ternary mixtures were investigated in oscillation modes by a hybrid rheometer (HR-2, TA Instruments, Milford, MA, USA) with parallel plates 25 mm in diameter. A small amplitude oscillatory shear test was carried out in a fixed strain of 3.125% with a constant frequency of 1 rad/s at a heating rate of 5 °C/min. The flexural strength of unmodified epoxy and cured modified epoxy thermosets were tested on a universal testing machine (AG-100KN, SHIMATSU, Kyoto, Japan) with specimens (80 mm × 10 mm × 4 mm) at a loading rate of 2 mm/min according to ISO178. The critical stress concentration factor (*K*_IC_) of the single edge notch bend (SENB) specimens (40 mm × 8 mm × 4 mm) was measured according to ASTM D5045-14 with a loading rate of 10 mm/min at the temperature of 23 °C. The no-notch impact strength of the cured epoxy specimens (80 mm × 10 mm × 4 mm) was determined by an impact tester (CEAST9050, INSTRON, Boston, MA, USA) according to ISO179. The glass translation temperature (*T*_g_) and flexural modulus of the cured epoxy specimens (35 mm × 7 mm × 2 mm) were analyzed under a bending mode (1 Hz) with an amplitude of 5 mm and a heating rate of 10 °C /min over a temperature range of 25–200 °C on a dynamic mechanical analysis (DMA, DMS6100, SII, Tokyo, Japan) according to ASTM D4092 and D4085. The fracture surfaces of the cured epoxy composites after impact tests were observed with a scanning electron microscope (SEM, S-4800, HITACHI, Tokyo, Japan). 

## 3. Results and Discussion

### 3.1. FT-IR Analysis on PTMG and Prepared Tetra-Carbamates

**Figure 2:**
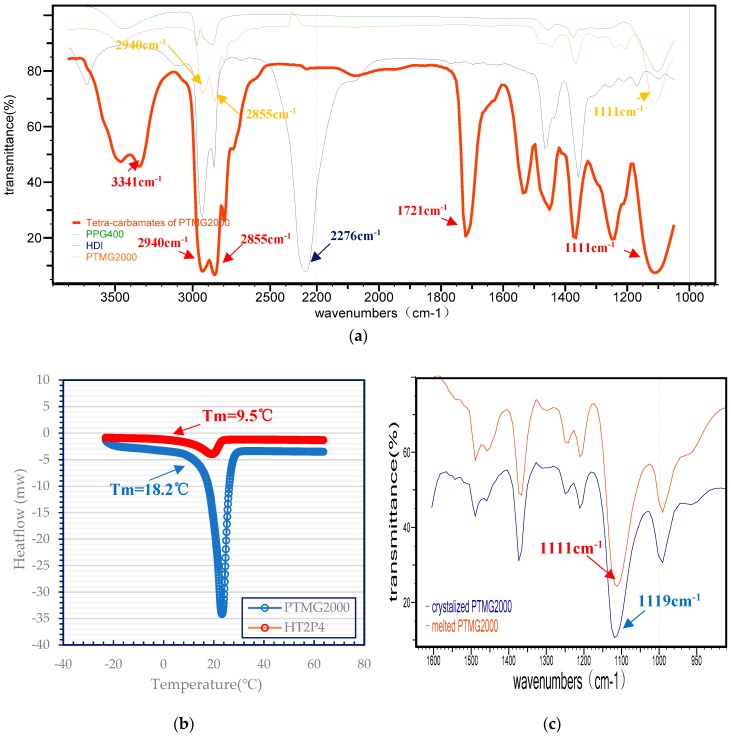
(**a**) FT-IR spectra of HDI, PTMG2000, PPG400 and HT2P4 at 25 °C; (**b**) DSC profile of PTMG2000 and HT2P4; (**c**) FT-IR spectra of crystalized PTMG2000 and melted PTMG2000, respectively.

The molecular structure of tetra-carbamates is shown in Figure 1. It was synthesized via the facile addition reaction of isocyanate with hydroxyl. An FT-IR Spectrophotometer was employed to record the infrared spectra of samples, and a DSC was used to study the thermal properties of samples. Comparing to FT-IR spectra of HDI, no characteristic absorption peak corresponding to isocyanate(–N=C=O) was observed at 2276 cm^−1^ in the FT-IR spectra of the HT2P4 sample. This indicates that the additional reactions of –N=C=O from HDI and hydroxy groups (–OH) from PTMG2000 and PPG400 were fully completed [18]. The strong IR absorptions at 3341 and 1721 cm^−1^ corresponding to the stretching vibration of N–H and C=O, respectively, indicated that there exist carbamate groups of –NHCOO– in the prepared HT2P4 molecules [18,19,20]. Meanwhile, the strong absorption peaks at 3442 and 1111 cm^−1^ corresponding to the stretching vibration of –OH and C–O, respectively, showed the existence of the hydroxyl groups and ether bonds in the molecular structure of prepared HT2P4 [4]. Furthermore, HT2P4 showed a slight phase change behavior as shown in Figure 2b. However, its melting temperature (~9.5 °C) was much lower than that of pure PTMG2000 (~18.2 °C), which is related to the poor crystallizaiton of the PTMG segment of HT2P4 attributed to the steric hindrance from the side carbamates and PPG chains. All results indicated that the HDI reacted completely with the PTMG and PPG to form PPG-terminated tetra-carbamates as shown in Figure 1. It is interesting to find that the absorbance position of C=O stretching is not observed at ~1683 cm^−1^ but at 1721 cm^−1^. Many researches have shown that the absorbance position of C=O stretching is always at 1683~1707 cm^−1^ due to the strong hydrogen bonding with –N–H composed in reported carbamates and polyurethane [19,20,21,22,23]. This indicates that the C=O in HT2P4 is non-hydrogen-bonded carbonyl. However, HT2P4 spectra show an absorption corresponding to intensively hydrogen-bonded N–H stretching vibrations (3341 cm^−1^) in Figure 2a [22]. It is speculated that hydrogen bonding happens between N–H and other acceptors, such as ether bonds of C–O–C from HT2P4. We rationalize this behavior on the basis of the wavenumber shift of the C–O– stretching vibration from 1119 cm^−1^ in the IR spectra of the crystalized PTMG2000 to 1111 cm^−1^ in the IR spectra of the melted PTMG2000 as shown in Figure 2c.

### 3.2. Rheological Analysis on Tetra-Carbamates/Epoxy Blends and the Tetra-Carbamates/Epoxy/TETA Curing System

**Figure 3:**
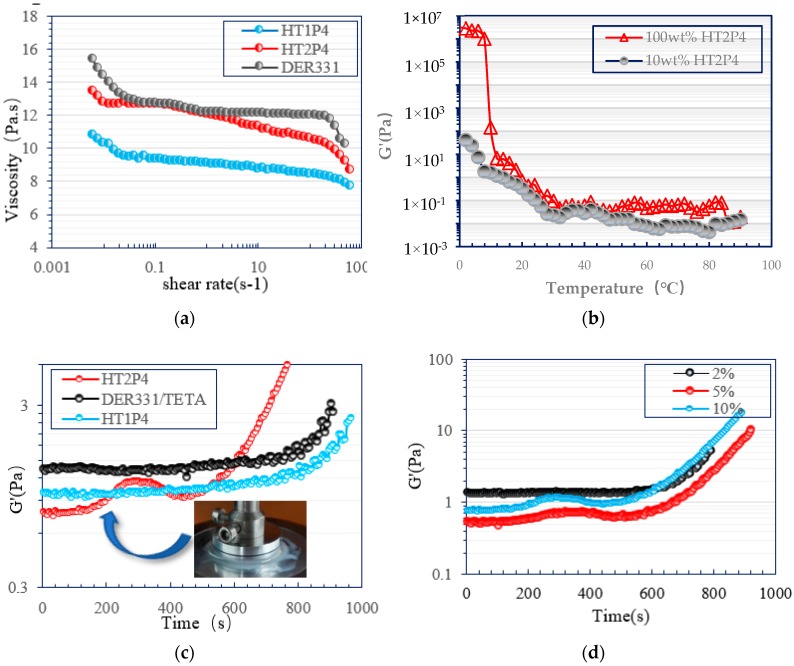
(**a**) Steady shear viscosity profiles of neat DER331, DER331/HT1P4 (10 wt %) and DER331/HT2P4 (10 wt %) as a function of shear rate at 25 °C; (**b**) Dynamic viscoelastic moduli vs. temperature by oscillatory shear for neat HT2P4, DER331/HT2P4 (10 wt %) blend at sweep cooling rate of 5 °C/min (strain = 3.125%, angular frequency = 1 rad/s, 25 mm parallel plate); (**c**) Dynamic viscoelastic moduli vs. time by oscillatory shear for neat DER331/TETA blend, DER331/TETA/HT1P4(10 wt %) blend, and DER331/TETA/HT2P4(10 wt %) blend at 60 °C (strain = 3.125%, angular frequency = 1 rad/s, 25 mm parallel plate); (**d**) Dynamic viscoelastic moduli vs. time by oscillatory shear for DER331/TETA/HT2P4 blends at different mass ratio of HT2P4 at 60 °C (strain = 3.125%, angular frequency = 1 rad/s, 25 mm parallel plate).

The effect of different tetra-carbamates additives on the viscosities of DER331 resin systems at 25 °C is shown in Figure 3a. Both modified epoxy systems with HT1P4 and HT2P4 at 10 wt % showed lower viscosities than the neat DER331 system, with shear rates varying from 0.00625 to 625 s^−1^ via a log-log scale. This is a distinct advantage of these additives when compared to the commercial toughening additives, which always result in the significant increase in viscosity of modified epoxy system, such as CTBN and BCs [7,11,12,13,14]. The viscosity of the epoxy system modified with HT1P4 is the lowest, which is attributed to the lower molecular weight of HT1P4. Understanding the interaction and miscibility between components is the key to obtaining high-performance polymer materials in the polymer industry [24,25,26]. The dynamic oscillation test is considered an acute method to characterize the underlying microstructure in dispersion systems which can be probed in the at-rest state without disruption [27,28,29,30]. The underlying microstructure of tetra-carbamates in neat epoxy and epoxy/amine blend systems was characterized by a rheometer through a small amplitude oscillatory shear test. Figure 3b shows the difference in dynamic storage modulus (G′) of neat HT2P4 and its blend with epoxy at 10% mass percentage as a function of temperature. The G′ of neat HT2P4 was observed to be higher than that of the modified epoxy resin with HT2P4. This indicates that the intermolecular interaction of HT2P4 was higher than the intramolecular interaction between HT2P4 and bisphenol-A epoxy molecules. This was also accordant with the low viscosity behavior of epoxy systems containing tetra-carbamates additives. 

The effect of different tetra-carbamates additives on the curing profile of the DER331/TETA system at 60 °C is shown in Figure 3c. The curing profile of the DER331/TETA system modified with HT1P4 was very close to that of the unmodified DER331/TETA system, but different from that of the modified DER331/TETA system with HT2P4. The general increase in the G′ for three various epoxy systems with time elapsing could be attributed to the increase in molecular weight caused by the curing reaction of epoxy resin and amine. The sharp increases in the G′, starting from ~600 s in the curing profiles of the unmodified epoxy system, and the modified epoxy system with HT1P4 were related to the formation of the crosslinked gel. However, another sharp increase in the G′ was observed at the early stage in the curing profile of the modified epoxy system with HT2P4 as shown in Figure 3c. It is speculated that the early sharp increase in the G′ was ascribed to the formation of some micro-structures in the epoxy matrix caused by the curing reaction. This is accordant with the change in the sample aspect from initial transparency to milky white during the oscillation test as shown in Figure 3c. Thus, it is proposed that the formation of the microstructures follows the mechanism of curing reaction-induced microphase separation. Furthermore, it is found that the early sharp increase in the G′ becomes less dramatic with the decrease in mass percentage of HT2P4 in modified epoxy systems, and even disappears at 2 wt % (Figure 3d). It is interesting to note that the tested sample with 2 wt % HT2P4 turned semitransparent. This aspect change could be interpreted on the basis of the formation of some microstructures whose sizes are too small to be detected by the rheometer. This behavior of the modified epoxy system with HT2P4 is different from that of the modified epoxy system with HT1P4. For epoxy systems modified with HT1P4, all samples showed the transparent and semitransparent aspects. 

### 3.3. Cured Properties of Epoxy/TETA Modified with the Additives of Tetra-Carbamates

**Figure 4:**
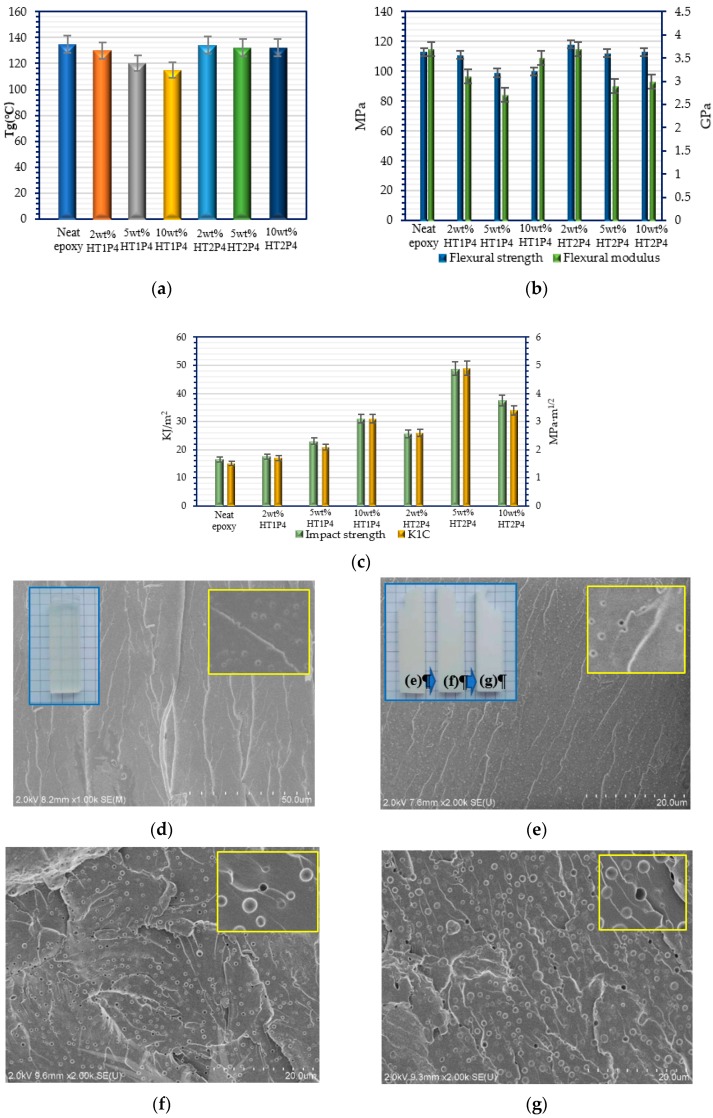
Cured properties of the cured unmodified epoxy, modified epoxy with HT1P4 and HT2P4 at 2, 5 and 10 wt %, respectively (**a**) *T*_g_; (**b**) Flexural strength and modulus; (**c**) Impact strength and *K*_IC_; (**d**) SEM for modified epoxy with 10 wt % HT1P4; (**e**) SEM for modified epoxy with 2 wt % HT2P4; (**f**) SEM for modified epoxy with 5 wt % HT2P4; (**g**) SEM for modified epoxy with 10 wt % HT2P4.

The cured properties of modified epoxy/TETA systems with HT1P4 and HT2P4 were characterized to study the effect of tetra-carbamates on the cured epoxy thermosets. Less than 10 wt % additives were employed in the DER331/TETA system considering the balance of cost and toughness improvement in practical applications. All modified epoxy systems showed as transparent liquids before the curing reaction. However, the cured aspects were observed differently according to the employed tetra-carbamates in epoxy systems. The modified epoxy systems with HT1P4 often showed the cured semitransparent aspects, which can be ascribed to the good miscibility of HT1P4 in epoxy systems. Microphase separation induced by curing reaction was limited in the epoxy system containing HT1P4, whereas phase separation was executed fully in the modified epoxy system with HT2P4, which resulted in the cured opaque aspects as shown in Figure 4e.

The effect of HT1P4 additive on the cured properties of modified epoxy systems is shown in Figure 4a–d. It can be seen that *T*_g_, flexural strength, and modulus of epoxy systems modified with HT1P4 decreased with the increase in the content of additives from 0 to 5 wt %, which can be ascribed to the internal plasticization of miscible HT1P4. The impact strengths and *K*_IC_ of the modified epoxy systems showed a slight increase accordingly. However, it is exciting to find that the flexural strength and modulus of epoxy systems modified with 10 wt % HT1P4 increased with little further reduction in *T*_g_. Compared to the unmodified epoxy system, they declined approximately 14% and 5% with a 15 °C reduction in *T*_g_, respectively. Meanwhile, it was observed that the modified epoxy systems exhibited significantly higher impact strength and *K*_IC_ than the unmodified epoxy systems. Impact strength increased 84.8% (16.5–31.0 kJ/m^2^) and *K*_IC_ increased 107% (1.5–3.1 MPa·m^1/2^). The reduction in *T*_g_ by HT1P4 was much lower than that by the commercial plasticizers such as PPG400 at high additive contents. It is speculated that phase separation of HT1P4 in an epoxy matrix reduced the residual HT1P4 in a cured epoxy matrix. Figure 4d shows the SEM micrographs of fracture surfaces of the modified epoxy resin with 10 wt % HT1P4 after impact testing. The rough fracture surface is presented, which contains a number of crack propagations and plastic deformation. Meanwhile, spherical particles and cavities of ~0.3 µm in diameter were observed uniformly dispersing in the continuous epoxy matrix. The particle size is consistent with those of researched typical multiphase systems which exhibit significant toughening performances via particle cavitation [17,31]. Therefore, the mechanism of toughening in this work can be explained by a phase separate mechanism, an in situ reinforcing and toughening mechanism, and a particle cavitation mechanism [32,33].

The effect of HT2P4 additives on the cured properties of modified epoxy systems is shown in Figure 4a–c,e–g. Figure 4e–g present the SEM micrographs of fracture surfaces of the cured modified epoxy systems after impact testing. A typical biphasic structure of micron sized particles dispersed in an epoxy matrix is exhibited in all fracture surfaces. The sizes of the particles and cavities appear to vary from ~0.5 to ~1.5 µm in diameter with the increase in mass percentages of HT2P4 from 2 to 10 wt %. It is noteworthy that the fracture surfaces of these modified epoxy systems showed rougher surfaces and more crack propagations and plastic deformation than those of researched typical multiphase systems [34,35,36]. Amounts of cracks and shear yielding due to plastic deformation around the particle cavitation can absorb fracture energy, and then improve the impact resistance of cured epoxy thermosets [37]. As shown in Figure 4a–c, the modified epoxy systems exhibited higher impact strength and *K*_IC_ than the unmodified epoxy polymers. Impact strength of the modified epoxy system with 5 wt % HT2P4 increased 195.8% (16.5–48.8 kJ/m^2^), and accordingly *K*_IC_ increased 226.7% (1.5–4.9 MPa·m^1/2^). This is a particular advantage of the tetra-carbamates when compared to the commercial additives such as CTBN. It was reported that the toughness was improved by 60% at a level of 10 wt % of CTBN [17]. The toughness improvement of modified epoxy systems with tetra-carbamates was also higher than those of the reported epoxy systems with triblocks and diblocks of PPO and PEO [31,35,38]. The strong interfacial bonding between separated tetra-carbamates microphases and the cured epoxy matrix is speculated to positively support the great dissipation of energy due to the hydrogen bonding between four carbamates groups of –NHCOO– and enough cured hydroxyl –OH within the epoxy matrix. Meanwhile, the *T*_g_ and flexural strength values of the modified epoxy systems remained nearly invariant irrespective of the content of HT2P4. This indicates that molecules of HT2P4 were hardly retained in the epoxy matrix during the curing process. The flexural modulus values of the modified epoxy systems slightly decreased with the increase in the mass percentage of HT2P4, which could be ascribed to the deformation of the epoxy matrix around the particle cavitation [39], whereas the reduction in *T*_g_ of the modified epoxy system with 5 wt % commercial CTBN was reported to be more than 15 °C [17].

## 4. Conclusions

PPG-terminated tetra-carbamates were synthesized as a new toughening additive for epoxy thermoset polymers. The modified epoxy systems with these additives exhibited lower steady viscosities than the unmodified epoxy resin, which indicates that they had a positive effect on the processing viscosity. The impact strength and *K*_IC_ of the modified epoxy systems with the relatively low concentration of prepared tetra-carbamates composed of PTMG segments with bigger molecular weight were significantly higher than those of unmodified epoxy polymer without sacrificing *T*_g_ and flexural strength. The toughening mechanism is supposed as a synergism combination among the phase separation mechanism, an in situ homogeneous toughening mechanism, and a particle cavitation mechanism. In conclusion, the prepared tetra-carbamates have excellent potential for use in the practical applications of epoxy resin considering the cost, processability, and desired toughness improvement.

## Figures and Tables

**Table 1:** Chemical components for the samples.

Sample Name	Isocyanate	Dihydric Alcohol A	Dihydric Alcohol B
HT1P4	HDI	PTMG650	PPG400
HT2P4	HDI	PTMG2000	PPG400
